# Predictive Factors of Neurological Complications and One-Month Mortality after Liver Transplantation

**DOI:** 10.3389/fneur.2014.00275

**Published:** 2014-12-17

**Authors:** Katherine A. Fu, Joseph DiNorcia, Linda Sher, Shamsha A. Velani, Shahrzad Akhtar, Laura A. Kalayjian, Nerses Sanossian

**Affiliations:** ^1^Keck School of Medicine, University of Southern California, Los Angeles, CA, USA; ^2^Department of Neurology, University of Southern California, Los Angeles, CA, USA; ^3^Department of Hepatobiliary, Pancreas and Abdominal Organ Transplant Surgery, University of Southern California, Los Angeles, CA, USA

**Keywords:** liver transplant, neurological complications, mortality

## Abstract

**Background:** Neurological complications are common after orthotopic liver transplantation (OLT). We aimed to characterize the risk factors associated with neurological complications and mortality among patients who underwent OLT in the post-model for end-stage liver disease (MELD) era.

**Methods:** In a retrospective review, we evaluated 227 consecutive patients at the Keck Hospital of the University of Southern California before and after OLT to define the type and frequency of and risk factors for neurological complications and mortality.

**Results:** Neurological complications were common (*n* = 98), with encephalopathy being most frequent (56.8%), followed by tremor (26.5%), hallucinations (11.2%), and seizure (8.2%). Factors associated with neurological complications after OLT included preoperative dialysis, hepatorenal syndrome, renal insufficiency, intra-operative dialysis, preoperative encephalopathy, preoperative mechanical ventilation, and infection. Preoperative infection was an independent predictor of neurological complications (OR 2.83, 1.47–5.44). One-month mortality was 8.8% and was independently associated with urgent re-transplant, preoperative intubation, and intra-operative arrhythmia.

**Conclusion:** Neurological complications are common in patients undergoing OLT in the post-MELD era, with encephalopathy being most frequent. An improved understanding of the risk factors related to both neurological complications and one-month mortality post-transplantation can better guide perioperative care and help improve outcomes among OLT patients.

## Introduction

Orthotopic liver transplantation (OLT) is the only definitive treatment for end-stage liver disease and is being performed with increasing frequency in the United States (1). Post-operative complications after OLT are a significant source of morbidity and mortality (2, 3), with neurological complications such as encephalopathy, akinetic mutism, seizures, and stroke reportedly occurring in 8% to as high as 75% of cases, often within 30 days of transplantation (4–7). Neurological complications occur more commonly after OLT than after other solid organ transplantations such as heart and kidney (8), and studies have suggested that these higher rates are related to preoperative hepatic encephalopathy, the unfavorable clinical condition of patients awaiting transplantation (e.g., malnutrition, renal insufficiency, hyponatremia, coagulopathy), and the complexity of the operation (9). Post-operatively, patients with neurological complications may become less compliant with immunosuppressive medications, increasing the risk of rejection and graft loss (10, 11). Research has demonstrated that patients with neurological complications after OLT have longer hospitalizations and increased rates of infection, re-transplantation, and mortality (12, 13).

Defining the risk factors for neurological complications and one-month mortality after OLT can help identify high-risk patients and guide perioperative care with the intention to minimize neurological complications, morbidity, and mortality. Identifying primary risk factors is particularly relevant given the high acuity recipients in the post-model for end-stage liver disease (MELD) era, as the MELD system prioritizes the allocation of organs to patients with the most severe liver disease (14). Studies have suggested that neurological complications can be attributed to factors such as cerebral infarction, a poorly functioning allograft, intracranial hemorrhage, infection, or immunosuppressant toxicity (15).

While complications after OLT have been described previously (15), risk factors for post-operative neurological complications and one-month mortality are not well-characterized. In this study, we defined the type and frequency of neurological complications in patients who underwent OLT in the post-MELD era. We identified hypothesis-generating pre-operative and intra-operative risk factors for neurological complications and one-month mortality to help improve future patient outcomes after OLT.

## Materials and Methods

### Dataset and study population

We reviewed the hospital records of 227 consecutive cases of adults who underwent OLT between July 18, 2003 and August 19, 2006 at the Keck Hospital of the University of Southern California. A standardized form was used to collect demographic, historical, surgical, laboratory and radiological data. Data was collected for the period beginning one week prior to transplant and extending to one month post-transplant. The Institutional Review Board approved the study.

Transplants were characterized as partial living donor or cadaveric and as first transplant, urgent re-transplant (performed ≤ one month from initial transplant), non-urgent re-transplant (performed > one month from initial transplant), transfusion free, and simultaneous liver-kidney transplant. MELD scores were calculated on the day of transplant and included exception points where applicable in cases of hepatocellular carcinoma. Operating room records were reviewed for intra-operative events, surgery duration, pressors used, and hypotension (defined as systolic blood pressure < 90 mmHg). For re-transplants, preoperative data, labs, and intra-operative records from the first transplant were recorded. Neurological complications were identified by chart review by the study’s investigators, including resident physicians and attending physicians in the Department of Neurology. These complications were then confirmed by the study’s multiple board certified neurologist investigators. These complications were defined as new neurological problems or a worsening of known neurological disorders in one month after OLT. Neurological complications were further characterized by pre-specified type and date of onset relative to OLT.

### Statistical analysis

An analysis was done to determine whether neurological complications post-OLT were associated with one-month mortality post-OLT. Univariate analyses of mortality and neurological complications after OLT were performed using Fisher’s Exact Test and Wilcoxon Two Sample Test where appropriate, using a *p*-value <0.05 threshold for significance. Using a *p* <0.2 threshold for significance, multivariate logistic regression analysis was performed controlling for potential risk factors for neurologic complications after OLT; these included medical co-morbidities such as dialysis and renal insufficiency, preoperative conditions such as encephalopathy and infection, and intra-operative events such as dialysis. Six cases were excluded from this analysis due to intra-operative deaths. A similar multivariate regression analysis was performed controlling for potential risk factors for one-month mortality following OLT, such as race, transplant type, medical co-morbidities, preoperative conditions and intra-operative events, again using a *p* < 0.2 threshold.

## Results

We identified 228 consecutive adult patients who underwent OLT. One patient record could not be found, thus 227 patients were included in the study. Demographic factors are listed in Table 1. The population was 37% female with a mean age of 52 years and mostly Hispanic (48.9%) or Caucasian (30.4%). The mean MELD score was 22 (range from 2 to 40). The most common liver diseases were hepatocellular carcinoma (20.7%), hepatitis C (19.8%), and alcohol-induced cirrhosis (15.9%). The majority of transplants were cadaveric (79.7%) and first-time transplants (94.3%).

**Table 1:** **Demographics of patients receiving liver transplant**.

Category	Characteristics	Entire study population, number (%) (*n* = 227)	No Neurological complications, number (%) (*n* = 129)	Neurological complications, number (%) (*n* = 98)
Gender	Male	142 (62.6)	74 (57.4)	63 (64.3)
Race	Hispanic	111 (48.9)	59 (45.7)	51 (52.0)
	Caucasian	69 (30.4)	41 (31.8)	25 (25.5)
	Asian	29 (12.8)	15 (11.6)	14 (14.3)
	African American	9 (4)	4 (3.1)	5 (5.1)
	Other	9 (4)	4 (3.1)	3 (3.1)
Donor type	Cadaveric (vs. partial living)	181 (79.7)	94 (72.9)	81 (82.7)
Transplant	First transplant	214 (94.3)	110 (85.3)	91 (92.9)
	Urgent re-transplant	8 (3.5)	4 (3.1)	4 (4.1)
	Non-urgent re-transplant	5 (2)	9 (7.0)	3 (3.1)
Co-transplant	None	212 (93.4)		
	Liver–kidney	15 (6.6)	10 (7.8)	4 (4.1)
Disease etiology	Hepatocellular Carcinoma	47 (20.7)	28 (21.7)	18 (18.4)
	Hepatitis C	45 (19.8)	36 (27.9)	29 (29.6)
	Alcohol	36 (15.9)	19 (14.7)	17 (17.3)
	Cryptogenic	16 (7)		
	Hepatitis B	14 (6.2)	11 (8.5)	3 (3.1)
	Autoimmune	10 (4.4)		
	Primary biliary cirrhosis, fulminant failure, other	6 (2.6)		
	Metastasis	3 (1.3)		
	Alpha 1 antitrypsin, amyloid, polycystic liver, cholangiocarcinoma, rejection, sclerosing cholangitis, Wilson’s	2 (0.9)		

### Neurological complications

The one-month incidence of neurological complications was 44.3% (98/221). Sixty-six patients had one neurological complication, 27 had two complications, and five had three complications. The most common complication was encephalopathy (53.06%), followed by tremor (27.55%) and seizure (8.16%). Neurological complications among patients during the first post-operative month following OLT are summarized in Table 2. Neurological complications were not associated with one-month mortality.

**Table 2:** **Neurologic complications during the first post-operative month after OLT**.

Complication	Cases (*n* = 98) number (%)	% of total population (227)
Encephalopathy	52 (53.1)	22.9
Tremor	26 (26.5)	11.5
Hallucination	11 (11.2)	4.8
Seizure	8 (8.2)	3.5
Headache	5 (5.1)	2.2
Intracerebral hemorrhage	5 (5.1)	2.2
Ischemic stroke	5 (5.1)	2.2
Myoclonus	3 (3.1)	1.3
Anxiety	2 (2.0)	0.9
Paresthesia	2 (2.0)	0.9
Dysarthria	2 (2.0)	0.9
Central pontine myelinolysis	1 (1.0)	0.04
Neuropathy	1 (1.0)	0.04
Intracranial infection	1 (1.0)	0.04
Cerebral edema	1 (1.0)	0.04
Subclinical status epilepticus	1 (1.0)	0.04
Leg twitching	1 (1.0)	0.04
Leg weakness	1 (1.0)	0.04
Viral myositis	1 (1.0)	0.04
Tinnitus	1 (1.0)	0.04
Posterior reversible leukoencephalopathy	1 (1.0)	0.04

Univariate analysis of risk factors for neurological complications is summarized in Tables 3 and 4. 95% confidence intervals (CI) for univariate analyses were calculated assuming normal distribution. Although the MELD score was greater in those with neurological complications, it was not statistically significant (20 vs. 24, *p* = 0.06). Medical co-morbidities, such as hepatorenal syndrome (OR 2.9, 0.87–2.91), renal insufficiency (OR 2.23, 0.93–2.17), and intra-operative dialysis (OR 1.97, 0.80–2.25) were also risk factors associated with a greater rate of neurological complications. Furthermore, preoperative use of pressors (OR 2.72, 0.71–3.36), intubation (OR 2.8, 0.78–3.15), and encephalopathy (OR 1.97, 0.86–2.09) were also factors associated with increased neurological complications based on the results of the univariate analysis.

**Table 3:** **Univariate analysis of risk factors for neurologic complications following OLT**.

Variable	OR	95% CI (*p*-value)
Medical comorbidity
Dialysis	2.11	0.80–2.40 (0.041)
Hepatorenal syndrome	2.9	0.87–2.91 (0.01)
Renal insufficiency	2.23	0.93–2.17 (0.006)
Preoperative conditions
Encephalopathy	1.97	0.86–2.09 (0.023)
Pressor use	2.72	0.71–3.36 (0.052)
Intubation	2.8	0.78–3.15 (0.028)
Infection^a^	2.83	0.98–2.52 (0.002)
Intra-operative events
Dialysis	1.98	0.80–2.25 (0.046)

*Variables with *p* < 0.2 are included*.

*^a^Significant on multivariate analysis*.

**Table 4:** **Univariate analysis of laboratory risk factors for neurologic complications after OLT**.

Variable	Mortality group (level ± SD)	Survival group (level ± SD)	*p*-Value
MELD score	23.51 ± 11.9	20.24 ± 10.88	0.056
Hemoglobin (g/dL)	10.72 ± 1.91	11.31 ± 2.13	0.03
PTT (s)	39.13 ± 10.64	36.23 ± 8.49	0.026
INR (IU)	1.73 ± 0.84	1.52 ± 0.56	0.06
Number of intra- operative pressors	1.36 ± 0.66	1.15 ± 0.75	0.027

*OLT: orthotopic liver transplantation; MELD: Model for end-stage liver disease; PTT: Partial thromboplastin time; INR: international normalized ratio. Laboratory values with *p* < 0.2 are included*.

On multivariate analysis, preoperative infection was the only independent predictor of neurological complications (OR 2.83, 1.49–5.53). There were a total of 50 patients who had preoperative infection, of which 32 (64%) had neurological complications. The most common neurological complications in this group were encephalopathy (56.25%) and tremor (21.88%). Exclusion of intra-operative deaths did not change the study results.

### Mortality

Overall, one-month mortality was 8.8%. The MELD score was greater in the mortality group, but not statistically significant (25 vs. 22, *p* = 0.181). Of the 20 deaths, six occurred intraoperatively (30%). Characteristics of the 14 mortalities in the study population are listed in Table 5. Factors associated with mortality based on results of the univariate analysis are listed in Tables 6 and 7. 95% CI for univariate analyses were calculated assuming normal distribution. Preoperative pressor use (OR 4.97, 0.78–5.19) and infection (OR 3.21, 0.78–3.15) as well as intraoperative cardiac arrest (OR 55.19, 1.00–32.63) and hypotension (OR 7.78, 0.94–6.34) were associated with greater mortality.

**Table 5:** **Characteristics of the 14 mortalities in the study population**.

Number	POD	Cause of death	Neurological complication
1	2	Cardiogenic shock	None
2	2	Sepsis	Encephalopathy
3	4	Pulmonary embolism	Stroke, Myoclonus
4	5	Cardiogenic shock	None
5	5	Herniation	Cerebral Edema
6	6	Cardiogenic shock	Lower extremity twitching
7	7	Cardiac arrest in later surgery	None
8	12	Sepsis	None
9	15	Cardiogenic shock	None
10	19	Herniation	Intracerebral Hemorrhage
11	21	Intracerebral hemorrhage	Intracerebral Hemorrhage
12	26	Sepsis	None
13	28	Asystole after seizure	Intracerebral Infection, Seizure
14	29	Sepsis	None

*POD, post-operative day*.

**Table 6:** **Univariate analysis of risk factors for one-month mortality following OLT**.

Variable	OR	95% CI (*p*-value)
**Transplant type**		
Urgent re-transplant*	12.69	0.87–10.40 (0.003)
Non-urgent re-transplant	3.48	0.55–5.38 (0.094)
Medical comorbidity		
Pre-existing neurologic disease	2.8	0.66–3.72 (0.071)
Recent surgery	7.56	0.46–12.54 (0.063)
**Preoperative conditions**		
Pressor use	4.97	0.78–5.19 (0.013)
Intubation^a^	4.5	0.81–4.58 (0.011)
Infection	3.21	0.78–3.51 (0.021)
**Intraoperative events**		
Arrhythmia^a^	4.97	0.78–5.19 (0.013)
Cardiac arrest	55.19	1.00–32.63 (<0.001)
Hypotension	7.78	0.94–6.34 (0.002)
Race	N/A	N/A (0.169)

*Variables with *p* < 0.2 are included*.

*^a^Significant on multivariate analysis*.

**Table 7:** **Univariate analysis of laboratory risk factors of one-month mortality following OLT**.

Variable	*Mortality group* (level ± SD)	*Survival group* (level ± SD)	*p*-Value
MELD score	24.75 ± 10.59	21.47 ± 11.42	0.181
Hemoglobin (g/dL)	9.94 ± 1.45	11.1 ± 2.09	0.024
PTT (s)	40.34 ± 11.21	37.41 ± 9.41	0.185
INR (IU)	1.99 ± 1.3	1.6 ± 0.7	0.151
Albumin (g/dL)	2.65 ± 0.7	3.11 ± 2.07	0.068
Number of intra- operative pressors	1.95 ± 1.1	1.19 ± 0.68	0.001

*OLT: orthotopic liver transplantation; MELD: Model for end-stage liver disease; PTT: Partial thromboplastin time; INR: international normalized ratio. Laboratory values with *p* < 0.2 are included*.

On multivariate analysis, independent predictors of one-month mortality included urgent re-transplant (OR 13.3, 2.9–55.53), preoperative intubation (OR 5.03, 1.54–13.14), and intraoperative arrhythmia (OR 5.75, 1.56–15.83).

## Discussion

The incidence of post-operative neurological complications after OLT in this study (44%) was comparable to the incidence reported in the literature of approximately 10–47% (1, 5). Previous studies have found that most neurological complications usually occur in the first 3 months after OLT (1). Similar to prior studies, we focused our study on the first post-operative month (4, 5).

Our results revealed high rates of neurological complications and mortality after OLT in the post-MELD era. These high rates of morbidity and mortality may reflect the selection of candidates with more advanced liver disease. The study population had a mean MELD score of 22, consistent with the reported mean MELD of 24 in the United Network for Organ Sharing (UNOS) MELD 2003–2006 database (16).

With improved survival after OLT due to better perioperative care, operative techniques, and immune suppressive therapy, there has been increasing awareness of neurological complications (17). The main neurological complications include encephalopathy, seizure, immunosuppressant-related neurotoxicity, and peripheral nerve damage (1, 18), and the distribution of neurological complications in this study is similar to prior reports, with encephalopathy being the most common (7, 9, 13, 19, 20). Established predictors of encephalopathy are alcoholism, metabolic dysregulation, MELD > 15, preoperative intubation, and non-elective liver transplant (21). Our findings demonstrated that neurological complications were not associated with one-month mortality. This lack of independent association suggests that encephalopathy, the most common neurological complication of this investigation, may present as a manifestation of other conditions such as infection or renal failure. Given these findings, the importance of separately considering encephalopathy when determining post-operative neurological events and mortality is worthy of additional study.

The primary aim of this investigation was to evaluate hypothesis-generating risk factors associated with post-operative neurological complications. We found that pre-operative infection was an independent predictor of such complications. Our study documented any infection in the week prior to surgery, including bacterial peritonitis and urinary tract infections. None of the patients who underwent OLT were septic at the time of surgery. Therefore, it appears that even mild or focal infections may be associated with an increased susceptibility to neurological events. This association is biologically plausible, as bacteria, viruses, and fungi can spread to the central nervous system just as they affect the abdomen, blood stream, respiratory tract, and other organ systems (22). Increases in incidence of encephalopathy and stroke were seen in patients with infection, possibly related to cerebral and vascular injury from the systemic inflammatory response. Systemic infection is particularly relevant as bacterial infections are common during the initial two months after transplantation and negatively affect both graft and patient survival (23). Transplant patients require high-dose immunosuppression, exacerbating the potential for acquiring infection and developing neurological complications post-operatively (11, 23).

In our analyses of hypothesis-generating risk factors for one-month mortality after OLT, re-transplantation within the first month was independently associated with one-month mortality. Urgent liver re-transplant is necessary when complications such as primary non-function or vascular thrombosis cause the allograft to fail (24). Our mortality rate of 50% in the first month for acute re-transplant is comparable to other studies, which cite a two-month mortality rate of 51% in patients who acutely undergo re-transplantation (25). Urgent re-transplantation has been shown to have much lower survival rates compared to overall liver re-transplantations, and our results corroborate this finding (26).

Pre-operative intubation also was an independent predictor of one-month mortality, a finding that has not been reported in prior studies of complications after OLT. One study has demonstrated that among patients who underwent emergency or elective living-donor liver transplantation (LDLT), those who underwent emergency LDLT had higher rates of pre-operative intubation, suggesting greater pre-transplant acuity and possibly poorer post-transplant outcome (27). Further, intraoperative arrhythmias were also independently associated with one-month mortality, corroborating studies that have suggested patients with atrial fibrillation may be at increased risk of mortality after liver transplantation (28, 29). Despite these few reports, risk factors associated with mortality after OLT have not been well characterized in the literature. Our results contribute to an improved understanding of the risk factors for post-transplant mortality in a vulnerable patient population.

While prior study populations were predominantly Caucasian, our study population was predominantly Hispanic. Studies have demonstrated poorer outcomes in minority populations, particularly African Americans (30). Some research has suggested that liver transplantation among Hispanics has excellent outcomes compared to non-Hispanics (31), while other studies have highlighted suboptimal outcomes among minority populations, including greater mortality rates (31–34). Our results demonstrated that African American and Hispanic patients had greater mortality than Caucasian patients. Though no factors were identified to explain these differences, possibilities include immunologic and genetic differences between recipients and donors.

The main limitation of this investigation is its retrospective design. It is possible that not all neurological findings were documented in the patient charts, resulting in an underestimation of some results. Our one-month follow-up may have missed neurological complications that occurred later in the post-operative course. Nevertheless, the findings contribute to a growing body of literature that examines neurological complications after OLT (12, 19, 28).

In summary, neurological complications after OLT are common in the post-MELD era, with encephalopathy being the most common complication. Infection was independently associated with the development of post-operative neurological complications, suggesting that these complications may be reduced by treating infection during the preoperative period. In addition, urgent re-transplant, preoperative intubation, and intraoperative arrhythmias were independent predictors of one-month mortality after OLT. Identifying hypothesis-generating risk factors for both the development of neurological complications and one-month mortality supports a better understanding of the population most at risk for adverse outcomes after transplantation.

Future studies might investigate the role of infection in neurological complications to better determine the pathophysiology involved in conditions such as encephalopathy and ischemic stroke among OLT patients. Similarly, factors this investigation revealed to be associated with one-month mortality after OLT, such as intraoperative arrhythmias, have been less well-characterized in the literature and warrant further investigation to clarify their impact on outcomes. An improved understanding of these hypothesis-generating risk factors for neurological complications and one-month mortality after liver transplantation ultimately may reduce adverse outcomes and improve graft and patient survival.

## Conflict of Interest Statement

The authors declare that the research was conducted in the absence of any commercial or financial relationships that could be construed as a potential conflict of interest.

## References

[1] Amodio P Biancardi A Montagnese S Angeli P Iannizzi P Cillo U Neurological complications after orthotopic liver transplantation. Dig Liver Dis (2007) 39(8):740–7 10.1016/j.dld.2007.05.004 17611177

[2] Mazariegos GV Molmenti EP Kramer DJ Early complications after orthotopic liver transplantation. Surg Clin North Am (1999) 79(1):109–29 10.1016/S0039-6109(05)70009-8 10073184

[3] Saner F Gu Y Minouchehr S Ilker K Fruhauf NR Paul A Neurological complications after cadaveric and living donor liver transplantation. J Neurol (2006) 253(5):612–7. 10.1007/s00415-006-0069-3 16511638

[4] Borhani Haghighi A Malekhoseini SA Bahramali E Firouzabadi N Salahi H Bahador A Neurological complications of first 100 orthotopic liver transplantation patients in southern Iran. Transplant Proc (2005) 37(7):3197–9. 10.1016/j.transproceed.2005.08.025 16213347

[5] Boin IF Falcao AE Luzo AC Cardoso AR Caruy CA Leonardi LS Analysis of neurologic complications within the first 30 days after orthotopic liver transplantation. Transplant Proc (2001) 33(7–8):3695–6 10.1016/S0041-1345(01)02507-6 11750574

[6] Dhar R Young GB Marotta P. Perioperative neurological complications after liver transplantation are best predicted by pre-transplant hepatic encephalopathy. Neurocrit Care (2008) 8(2):253–8. 10.1007/s12028-007-9020-4 17928960

[7] Ghaus N Bohlega S Rezeig M Neurological complications in liver transplantation. J Neurol (2001) 248(12):1042–8 10.1007/s004150170023 12013580

[8] Dhar R Human T Central nervous system complications after transplantation. Neurol Clin (2011) 29(4):943–72 10.1016/j.ncl.2011.07.002 22032668

[9] Bronster DJ Emre S Boccagni P Sheiner PA Schwartz ME Miller CM. Central nervous system complications in liver transplant recipients – incidence, timing, and long-term follow-up. Clin Transplant (2000) 14(1):1–7. 10.1034/j.1399-0012.2000.140101.x 10693627

[10] Bechstein WO. Neurotoxicity of calcineurin inhibitors: impact and clinical management. Transpl Int (2000) 13(5):313–26. 10.1111/j.1432-2277.2000.tb01004.x 11052266

[11] Tolou-Ghamari Z. Nephro and neurotoxicity of calcineurin inhibitors and mechanisms of rejections: a review on tacrolimus and cyclosporin in organ transplantation. J Nephropathol (2012) 1(1):23–30. 10.5812/jnp.6 24475383 PMC3886167

[12] Kim BS Lee SG Hwang S Park KM Kim KH Ahn CS Neurologic complications in adult living donor liver transplant recipients. Clin Transplant (2007) 21(4):544–7. 10.1111/j.1399-0012.2007.00687.x 17645717

[13] Lewis MB Howdle PD Neurologic complications of liver transplantation in adults. Neurology (2003) 61(9):1174–8 10.1212/01.WNL.0000089487.42870.C6 14610116

[14] Brown RS Jr Kumar KS Russo MW Kinkhabwala M Rudow DL Harren P Model for end-stage liver disease and Child-Turcotte-Pugh score as predictors of pretransplantation disease severity, posttransplantation outcome, and resource utilization in United Network for Organ Sharing status 2A patients. Liver Transpl (2002) 8(3):278–84. 10.1053/jlts.2002.31340 11910574

[15] Campagna F Biancardi A Cillo U Gatta A Amodio P. Neurocognitive-neurological complications of liver transplantation: a review. Metab Brain Dis (2010) 25(1):115–24. 10.1007/s11011-010-9183-0 20204483

[16] 2006 Annual Report of the U.S. Organ Procurement and Transplantation Network and the Scientific Registry of Transplant Recipients: Transplant Data 1996–2005. Health Resources and Services Administration, Healthcare Systems Bureau, Division of Transplantation, Rockville, MD (2006). Available from: http://www.srtr.org/annual_reports/archives/2006/2006_Annual_Report/default.htm

[17] Saner FH Sotiropoulos GC Radtke A Fouzas I Molmenti EP Nadalin S Intensive care unit management of liver transplant patients: a formidable challenge for the intensivist. Transplant Proc (2008) 40(9):3206–8. 10.1016/j.transproceed.2008.08.069 19010236

[18] Stracciari A Guarino M Neuropsychiatric complications of liver transplantation. Metab Brain Dis (2001) 16(1–2):3–11 10.1023/A:1011698526025 11726086

[19] Saner FH Gensicke J Olde Damink SW Pavlakovic G Treckmann J Dammann M Neurologic complications in adult living donor liver transplant patients: an underestimated factor? J Neurol (2010) 257(2):253–8. 10.1007/s00415-009-5303-3 19727899 PMC2910891

[20] Saner FH Sotiropoulos GC Gu Y Paul A Radtke A Gensicke J Severe neurological events following liver transplantation. Arch Med Res (2007) 38(1):75–9 10.1016/j.arcmed.2006.07.005 17174727

[21] Kanwal F Chen D Ting L Gornbein J Saab S Durazo F A model to predict the development of mental status changes of unclear cause after liver transplantation. Liver Transpl (2003) 9(12):1312–9. 10.1016/j.lts.2003.09.023 14625832

[22] Feltracco P Barbieri S Furnari M Milevoj M Rizzi S Galligioni H Central nervous system infectious complications early after liver transplantation. Transplant Proc (2010) 42(4):1216–22. 10.1016/j.transproceed.2010.03.108 20534265

[23] Kim SI. Bacterial infection after liver transplantation. World J Gastroenterol (2014) 20(20):6211–20. 10.3748/wjg.v20.i20.6211 24876741 PMC4033458

[24] Bellido CB Martinez JM Gomez LM Artacho GS Diez-Canedo JS Pulido LB Indications for and survival after liver retransplantation. Transplant Proc (2010) 42(2):637–40 10.1016/j.transproceed.2010.02.017 20304211

[25] Ferraz-Neto BH Zurstrassen MP Hidalgo R Rezende MB Meira-Filho SP Pandullo FL Results of urgent liver retransplantation in the state of Sao Paulo, Brazil. Transplant Proc (2006) 38(6):1911–2. 10.1016/j.transproceed.2006.06.073 16908320

[26] Gustafsson BI Backman L Friman S Herlenius G Lindner P Mjornstedt L Retransplantation of the liver. Transplant Proc (2006) 38(5):1438–9 10.1016/j.transproceed.2006.02.120 16797326

[27] Takeda K Tanaka K Kumamoto T Nojiri K Mori R Taniguchi K Emergency versus elective living-donor liver transplantation: a comparison of a single center analysis. Surg Today (2012) 42(5):453–9. 10.1007/s00595-011-0040-5 22116395 PMC7101615

[28] Vannucci A Rathor R Vachharajani N Chapman W Kangrga I. Atrial fibrillation in patients undergoing liver transplantation-a single-center experience. Transplant Proc (2014) 46(5):1432–7. 10.1016/j.transproceed.2014.02.020 24935310

[29] Bargehr J Trejo-Gutierrez JF Rosser BG Patel T Yataco ML Pungpapong S Liver transplantation in patients with atrial fibrillation. Transplant Proc (2013) 45(6):2302–6. 10.1016/j.transproceed.2013.02.130 23953542

[30] Callender CO Cherikh WS Miles PV Hermesch A Maddox G Nash J Blacks as donors for transplantation: suboptimal outcomes overcome by transplantation into other minorities. Transplant Proc (2008) 40(4):995–1000. 10.1016/j.transproceed.2008.03.063 18555098

[31] Mejia A Halff GA Esterl R Cigarroa F Speeg KV Villarreal R Outcome of liver transplantation in Hispanics versus non-Hispanics: is there a difference? Transplant Proc (2002) 34(4):1236–8 10.1016/S0041-1345(02)02799-9 12072326

[32] Nair S Eustace J Thuluvath PJ. Effect of race on outcome of orthotopic liver transplantation: a cohort study. Lancet (2002) 359(9303):287–93. 10.1016/S0140-6736(02)07494-9 11830194

[33] Mathur AK Schaubel DE Gong Q Guidinger MK Merion RM. Racial and ethnic disparities in access to liver transplantation. Liver Transpl (2010) 16(9):1033–40. 10.1002/lt.22108 20818740 PMC2936696

[34] Mathur AK Schaubel DE Zhang H Guidinger MK Merion RM. Disparities in liver transplantation: the association between donor quality and recipient race/ethnicity and sex. Transplantation (2014) 97(8):862–9. 10.1097/01.tp.0000438634.44461.67 24345895 PMC4293640

